# Unilateral deafness in children affects development of multi-modal modulation and default mode networks

**DOI:** 10.3389/fnhum.2014.00164

**Published:** 2014-03-25

**Authors:** Vincent J. Schmithorst, Elena Plante, Scott Holland

**Affiliations:** ^1^Department of Radiology, Cincinnati Children’s Hospital Medical Center, CincinnatiOH, USA; ^2^Department of Radiology, Children’s Hospital of Pittsburgh of UPMC, PittsburghPA, USA; ^3^Department of Speech, Language, and Hearing Sciences, The University of Arizona, TucsonAZ, USA

**Keywords:** functional MRI, default mode network, unilateral hearing loss, multi-modal processing, children

## Abstract

Monaural auditory input due to congenital or acquired unilateral hearing loss (UHL) may have neurobiological effects on the developing brain. Using functional magnetic resonance imaging (fMRI), we investigated the effect of UHL on the development of functional brain networks used for cross-modal processing. Children ages 7–12 with moderate or greater unilateral hearing loss of sensorineural origin (UHL-SN; *N* = 21) and normal-hearing controls (*N* = 23) performed an fMRI-compatible adaptation of the Token Test involving listening to a sentence such as “touched the small green circle and the large blue square” and simultaneously viewing an arrow touching colored shapes on a video. Children with right or severe-to-profound UHL-SN displayed smaller activation in a region encompassing the right inferior temporal, middle temporal, and middle occipital gyrus (BA 19/37/39), evidencing differences due to monaural hearing in cross-modal modulation of the visual processing pathway. Children with UHL-SN displayed increased activation in the left posterior superior temporal gyrus, likely the result either of more effortful low-level processing of auditory stimuli or differences in cross-modal modulation of the auditory processing pathway. Additionally, children with UHL-SN displayed reduced deactivation of anterior and posterior regions of the default mode network. Results suggest that monaural hearing affects the development of brain networks related to cross-modal sensory processing and the regulation of the default network during processing of spoken language.

## INTRODUCTION

Unilateral hearing loss (UHL) is quite prevalent in children. While incidence estimates in newborns are only on the order of 1 per 1000 (Watkin and Baldwin, 1999; Dalzell et al., 2000), prevalence estimates in school-aged children are on the order of five to ten percent as those with acquired loss are added to the ranks (Niskar et al., 1998). Additionally, prevalence of UHL in newborns may be underestimated due to factors such as lack of appropriate follow-up after newborn hearing screens. In the auditory domain, UHL primarily impacts sound localization ability and recognition of speech in noise. In individuals with binaural hearing, sounds differ in relative amplitude and phase between the ears as a function of sound location. These differences are processed in the superior olivary complex (SOC) in the brainstem to yield information on sound localization. These inter-aural differences are not available for individuals with UHL and thus these individuals suffer from impaired sound localization ability. Children with UHL also suffer deficits in speech recognition in noise (Bess et al., 1986; Sargent et al., 2001; Ruscetta et al., 2005) due to the lack of binaural “squelch”: the ability to separate signal from noise when the signal and noise come from different locations, producing intensity, and temporal differences in the two ears.

However, UHL may affect brain development beyond the auditory system, as subtle changes in auditory experience can have an important impact on the types of experiences that shape brain development. For example, normal-hearing children will look toward the source of an interesting sound, and be able to pair the visual information with the heard sound. Children with UHL, however, will have far fewer opportunities, as they have poor sound localization. Even if they should happen to be looking in the appropriate direction by chance, they still may have difficulty associating the sound with the correct visual stimulus. Therefore, subtle changes in how children with UHL experience the auditory landscape (e.g., lack of information about sound localization) may impact development of brain regions and networks utilized for cross-modal modulation of auditory response in the presence of visual stimuli and visual response in the presence of auditory stimuli.

Deficits in cross-modal modulation may also impact cognitive function; as some evidence also suggests deficits experienced by children with UHL extend into cognitive domains. Children with UHL not only show difficulties in speech and language, but also exhibit academic, behavioral, and psychosocial deficits (Bess and Tharpe, 1986; Bovo et al., 1988; Bess et al., 1998; Lieu, 2004; Lieu et al., 2010, 2012). It should be noted that in these studies, either age at onset at hearing loss was not ascertained or a large proportion of children were identified with UHL at age 5 or later. This makes it probable a large proportion of these children had acquired (rather than congenital) UHL, and demonstrates that acquired as well as congenital UHL is a risk factor for speech, language, and behavioral deficits. Why this spectrum of behavioral sequelae should occur for children who have normal auditory input at least monaurally is not understood. There is currently a paucity of research into how auditory-visual and cognitive processes may be affected by sensory deficits such as those experienced by children with UHL, despite the large prevalence of UHL in children. Such information is crucial for tailoring and optimizing management strategies.

In this study, we wished to investigate processing of spoken language simultaneous with a relevant visual stimulus in children with unilateral hearing loss of sensorineural origin (UHL-SN). We developed a novel magnetic resonance imaging (MRI)-compatible modified “Token Test” (De Renzi and Vignolo, 1962) in which the child sees an arrow move from one token (shape) on the screen to another one. The screen is filled with eight shapes of varying colors, sizes, and types. The child hears a sentence such as “touched the small green circle and the large blue square” and presses a button if the sentence matches what was seen on the screen. Our primary hypothesis is that functional activation of brain regions typically affected by cross-modal processing (e.g., primary and secondary auditory and visual processing regions) will differ for children with UHL-SN compared to their normal-hearing peers. As an exploratory investigation, we also hypothesized possible differences in brain regions recruited for higher-order cognitive function.

## MATERIALS AND METHODS

Approval was obtained for this study from the Institutional Review Board at Cincinnati Children’s Hospital Medical Center (CCHMC). Informed consent was obtained from one parent of all participants, with assent obtained from participants 8 years of age or older. Data was acquired from 2006 until 2011.

### PARTICIPANTS

Participants with UHL-SN between 7 and 12 years of age (*N* = 21; 11 with left UHL-SN, 10 with right UHL-SN; 5 with moderate or moderately severe UHL-SN, 16 with severe-to-profound UHL-SN, see **Table 1** for complete details) were either referred from the Audiology Clinic at CCHMC, or were recruited via flyers placed at CCHMC main and satellite locations. An attempt was made to restrict inclusion to participants with hearing loss of 2 years or greater duration, as reorganization of auditory pathways is complete after approximately 2 years (Vasama et al., 1995; Scheffler et al., 1998; Tschopp et al., 2000). Accordingly, the duration of hearing loss was verified as 2 years or greater for 17 of the participants with UHL-SN; however, duration was unknown for four participants. Participants with mixed or conductive loss were excluded. Normal-hearing controls (*N* = 23) were recruited via flyers. Exclusion criteria consisted of standard MRI exclusion criteria (e.g., metallic implants, orthodontic braces, etc.); non-native or non-monolingual English speaker; any history of head trauma, attention deficit hyperactivity disorder (ADHD), pervasive developmental disorder (PDD), or autism; active otologic disease or history of ear surgery (excluding tympanostomy tubes); any history of infections (e.g., meningitis or cytomegalovirus); or score less than 95% on the Northwestern University – Children’s Perception of Speech (NU-CHIPS; presented via soundfield).

**Table 1:** Audiologic information on all participants with unilateral hearing loss of sensorineural origin.

Subject	Age (years)	Gender	Side of hearing loss	Duration of hearing loss	Severity	PTA
UL01	7.3	M	Left	> = 2 Years	Severe-profound	97
UL04	10.1	M	Left	> = 2 Years	Severe-profound	100
UL05	10.2	F	Left	> = 2 Years	Severe-profound	93
UL06	9.0	M	Left	Unknown	Severe-profound	120
UL11	8.1	M	Left	> = 2 Years	Moderate	50
UL13	10.7	F	Left	Unknown	Moderate	43
UL14	11.2	M	Left	> = 2 Years	Moderate	40
UL15	7.2	F	Left	> = 2 Years	Severe-profound	93
UL16	9.2	M	Left	> = 2 Years	Moderate	45
UL17	7.4	M	Left	> = 2 Years	Severe-profound	120
UL19	9.8	F	Left	> = 2 Years	Moderately severe	70
UR01	9.0	F	Right	> = 2 Years	Severe-profound	110
UR02	11.6	M	Right	> = 2 Years	Severe-profound	95
UR03	9.5	M	Right	Unknown	Severe-profound	107
UR04	7.8	M	Right	> = 2 Years	Severe-profound	107
UR06	8.6	F	Right	> = 2 Years	Severe-profound	120
UR08	10.9	M	Right	Unknown	Severe-profound	102
UR09	7.3	F	Right	> = 2 Years	Severe-profound	100
UR12	10.1	F	Right	> = 2 Years	Severe-profound	103
UR14	9.4	M	Right	> = 2 Years	Severe-profound	120
UR15	9.2	M	Right	> = 2 Years	Severe-profound	92

Using standard pure-tone audiometry (pure-tone average at frequencies 500, 1000, and 2000 Hz), we verified that all participants with UHL-SN had normal-hearing (< = 20 dB HL) in the good ear and sensorineural hearing loss of at least moderate severity (> = 40 dB HL) in the impaired ear. All normal-hearing participants had < = 15 dB HL in both ears. There was no difference in the gender composition of the two groups (see **Table 2** for demographic and test data for the participants). Normal levels of cognitive function were verified via the Wechsler Full-Scale Intelligence Scale for Children (WISC-IV). The normal-hearing cohort had significantly higher levels of cognitive function on the WISC-IV full-scale IQ (see **Table 2**).

**Table 2:** Demographic information, WISC-IV Full-Scale IQ, type of scanner used, and performance on four tests typically used to test for auditory processing disorder in children for the normal-hearing children in the study and the children with unilateral hearing loss (mean ± std).

	Normal-hearing	UHL	*p*
Sex	13F, 10M	8F, 13M	0.36
Age (years)	9.7 ± 1.48	9.2 ± 1.73	0.34
Full-scale IQ	114.2 ± 9.95	105.2 ± 10.05	0.005
Scanner (siemens, philips)	14, 9	9, 12	0.23
SCAN-C filtered words (#correct out of 40 possible)	33.0 ± 4.47	32.4 ± 4.30	0.668
40% Time-compressed sentences (#correct out of 60 possible)	57.9 ± 3.32	58.2 ± 3.09	0.776
60% Time-compressed sentences (#correct out of 60 possible)	52.7 ± 3.96	51.5 ± 7.63	0.536
BKBSIN sentences (SNR loss relative to normative data)	0.6 ± 1.25	1.8 ± 1.74	0.013

### AUDITORY TESTING

Normal pure-tone audiometry results indicated normal unilateral (in the UHL-SN) or bilateral hearing reception (in the control group). The requirement of high scores on the NU-CHIPS indicated normal perception of speech, regardless of the presence of UHL. In addition, participants completed four tests of higher-order auditory function, which are often included in batteries designed to diagnose auditory processing disorder (APD) in children, in a soundproof audiometric booth at CCHMC. Of the four auditory tests, a significant difference in performance was found only on the speech-in-noise test, in which UHL-SN children performed significantly worse compared to normal-hearing children. The tests are described individually below and test results are provided in **Table 2**.

#### Filtered words

The test stimuli consist of one syllable words that have been low-pass filtered at 750 Hz with a roll-off of 30 dB per octave. Twenty words are administered to each ear monaurally (for UHL children, all words are administered to the good ear). This test is a subtest of the SCAN3:C test for APD in children (Keith, 2009).

#### Time-compressed sentences

The test stimuli consist of two lists of 10 sentences with 40% time compression, and two lists of 10 sentences with 60% time compression (Beasley and Freeman, 1977; Keith, 2002). The sentences were taken from the Manchester University Test A, modified for word familiarity in the United States. In scoring, three points are given for each sentence repeated correctly, and one point deducted for each section of the sentence (subject, object, or predicate) misinterpreted or not heard.

#### Bamford–Kowal–Bench Speech-in-Noise

Stimuli consist of the Bamford–Kowal–Bench (BKB) sentences (Bench et al., 1979) spoken by a male talker in four-talker babble at various SNR levels ranging from +21 dB to -6 dB. The test is modified for use in children from a previously developed speech-in-noise test (QuickSIN Speech-in-Noise test, Etymotic Research, 2001). The test contains 18 list pairs, of which the first eight were used for this test. Three or four key words in each sentence are scored as correct or incorrect. Results of the two lists are averaged and compared with normative data to obtain the SNR loss, defined as the increase in SNR from normative performance required to obtain 50% correct words in sentences.

### MRI SCANNING PROCEDURES

All scans were acquired either on a Siemens 3T Trio system or on a Philips 3T Achieva system [distribution of scans provided in **Table 2**; this did not vary between UHL and normal-hearing groups (*p* > 0.2, chi-squared)]. Auditory stimuli were presented through a custom-built MRI-compatible audio system using ER-30 headphones (Etymotic Research, Elk Grove Village, IL, USA) which provides a background noise level of < 10 dB SPL. The functional magnetic resonance imaging (fMRI) paradigm consisted of an adaptation of the “Token Test” (De Renzi and Vignolo, 1962) appropriate for children undergoing fMRI scanning. In this version of the classic receptive language test, an orange arrow moves from one “token” (a completely filled-in shape) on the video screen to another token. Simultaneously, the participant hears a sentence such as “touched the small green square and the large blue circle.” The participant was instructed to respond by button press if the presented auditory sentence matched what was seen on the video screen. Two classes of sentences were used to contrast simple receptive processing and processing of complex syntax. Simple sentences contained the co-ordinating conjunction “and” and named no more than two tokens in a single sentence. Complex sentences contained either the temporal term “before” or “after” and also named a maximum of two tokens in a single sentence. During the control trials, the visual stimulus was the same but the audio stimulus consisted of a continuous 440 Hz tone. The control stimulus was chosen in order to control for visual, motor, and sub-lexical auditory processing. Completely silent trials were not included, as that would unacceptably lengthen the total paradigm duration beyond what children 7 years old can typically tolerate, and would yield minimal additional information. Stimuli were presented monaurally to the good ear for the UHL-SN cohort and monaurally for the normal-hearing cohort; choice of ear was counterbalanced across participants.

A silent-gradient acquisition technique was employed to eliminate interference from the scanner gradients during stimulus presentation (Schmithorst and Holland, 2004). This is an important consideration for performing fMRI paradigms on hearing-impaired populations. The stimulus was presented during a 5 s completely silent interval. Six seconds of scanning followed (three acquisitions with a 2 second repetition time), for a time per stimulus of 11 s. Thirteen control trials, thirteen “simple” sentence trials, and thirteen “complex” trials were presented, for a total scan time of 7:09. Stimuli were presented using Presentation Software (Neurobehavioral Systems Inc., Albany, CA, USA). Stimulus order was randomized at runtime. fMRI scan parameters were: TR = 2000 ms, TE = 38 ms, FOV = 24 × 24 cm, matrix = 64 × 64, SENSE factor = 2, slice thickness = 5 mm, 25 slices acquired covering the whole brain. For anatomical coregistration, 3-D whole-brain MP-RAGE T1-weighted anatomical scans were also acquired (scan parameters: TR = 8 ms, TE = 3.71 ms, matrix = 256 × 256 × 180, resolution = 0.977 mm × 0.977 mm × 1 mm).

### DATA ANALYSIS

Due to the non-standard nature of the data acquisition, data was processed using in-house routines written in IDL (Exelis, Boulder, CO, USA).

#### First level analysis

Since the longitudinal relaxation of spins differs for the first, second, and third frame after each silent period, the frames were grouped according to first, second, or third scan after the silent period and analyzed separately. Motion correction was performed using a pyramid iterative algorithm (Thevenaz et al., 1998), which was repeated using each set (triplet) of frames as references. An intensity-based cost function was computed (Szaflarski et al., 2006) and the best set of reference scans were selected according to which yielded the minimum overall cost. Frames were “scrubbed” from analysis if the cost function exceeded a threshold for which motion was apparent via visual inspection (Szaflarski et al., 2006). Datasets were transformed into stereotaxic space using landmarks from the T1-weighted anatomical images. A general linear model was performed for the contrasts of all speech vs. control, simple speech vs. control, complex speech vs. control, with a linear function added to the design matrix as a covariate of no interest to account for possible scanner drift. Magnitudes and variances of functional contrast were combined across frame groups to yield a total *T*-score.

#### Second level analysis

In the second level analysis, the following steps were performed:

(1) It was desired to restrict group comparisons to regions activated or deactivated in either group. Therefore, one-sample *T*-tests were performed on the UHL-SN and normal-hearing cohorts separately, in order to find voxels with significant activation or deactivation in either group. Results were spatially filtered with σ = 4 mm. An intensity threshold of *Z* = 7.0 and spatial extent threshold of 65 voxels was used, shown to correspond to a family-wise-error (FWE) corrected *p* < 0.01 via Monte Carlo simulation (Ledberg et al., 1998); intrinsic spatial smoothness was estimated (necessary to avoid bias in the FWE estimates) via the construction of “noise images” [detailed in (Ledberg et al., 1998)]. Subsequent analyses were performed on the union of voxels with significant activation/deactivation in either group.

(2) On the subset of voxels found from step 1, a GLM was performed with UHL-SN status (coded as a dummy variable) as the variable of interest and age, sex, full-scale IQ, scanner, square root of the number of retained frames, and side of presentation as covariates of no interest. [We note that this analysis does not suffer from the possible problem of circularity or “double-dipping” (Kriegeskorte et al., 2009; Vul and Pashler, 2012) as the statistical procedure used to select the region of interest (ROI) is distinct from the statistical test used on the selected voxels]. Results were spatially filtered with σ = 4 mm. An intensity threshold of *Z* = 5.0 and spatial extent threshold of 60 voxels was used, shown to correspond to a FWE corrected *p* < 0.05 (the intensity and spatial extent thresholds are less than in the analysis in step 1 since fewer voxels are included).

#### Post hoc analyses

*Post hoc* analyses were conducted using the ROIs containing voxels found to exhibit significant differences between normal-hearing and UHL-SN children (from step 2) for the contrast of all sentences vs. control. The residual variance of scans acquired on the Siemens scanner vs. scans acquired on the Philips scanner was compared, to ensure that no bias resulted from combining data across scanner platforms. We also desired to investigate whether there was an effect of side of hearing loss on the activation differences between children with UHL-SN and normal-hearing children seen in the left posterior superior temporal gyrus. Accordingly, a GLM was performed with the average activation across that region as the dependent variable; with the side of hearing loss as the variable of interest; and with age, sex, IQ, scanner, and the motion parameter as covariates of no interest.

#### Additional analyses involving subpopulations

For the contrast of all speech vs. control, additional second-level analyses were performed according to the previously described procedure (steps 1 and 2), due to the slight heterogeneity of the population with UHL-SN. Separate analyses were performed, comparing normal-hearing children with subsets of (a) only children with severe-to-profound UHL-SN; (b) only children with verified duration of hearing loss greater than 2 years; (c) only children with left UHL-SN; (d) only children with right UHL-SN. An additional analysis compared children with left USNHL to children with right USNHL.

## RESULTS

There was no significant difference in task performance for the simple or complex sentences, the overall performance (all sentences), or the # of frames discarded due to motion (**Table 3**). The normal-hearing children outperformed the UHL-SN cohort for the complex sentences at a trend level (*p* < 0.1).

**Table 3:** In-scanner task performance for simple sentences (13 trials), complex sentences (13 trials), and all sentences (26 trials); and square root of the # of retained frames; for the normal-hearing children in the study and the children with unilateral hearing loss (mean ± std).

	Normal-hearing	UHL	*p*
Simple sentences	10.3 ± 1.64	9.7 + /-2.22	0.28
Complex sentences	10.8 ± 2.15	9.4 ± 3.01	0.09
All sentences	21.1 ± 3.44	19.1 ± 4.83	0.12
Sqrt # retained frames	9.6 ± 1.33	9.8 ± 1.02	0.56

### OVERALL ACTIVATION/DEACTIVATION

Activation was seen (data not shown) in the posterior superior temporal gyrus bilaterally (Wernicke’s area and its RH homolog), the left inferior frontal gyrus (Broca’s area), and the middle occipital gyrus bilaterally (BA 19/37/39). Deactivation was seen (data not shown) in the posterior cingulate/precuneus [the posterior aspect of the default mode network (DMN)], medial prefrontal and orbitofrontal regions (anterior DMN regions), the right primary auditory cortex (likely related to greater activation during the control task due to hearing the 440 Hz tone) and in precentral and post-central regions (related to the greater motor activity during the control task).

### REGIONS WITH SMALLER ACTIVATION IN CHILDREN WITH UHL

**FIGURE 1:**
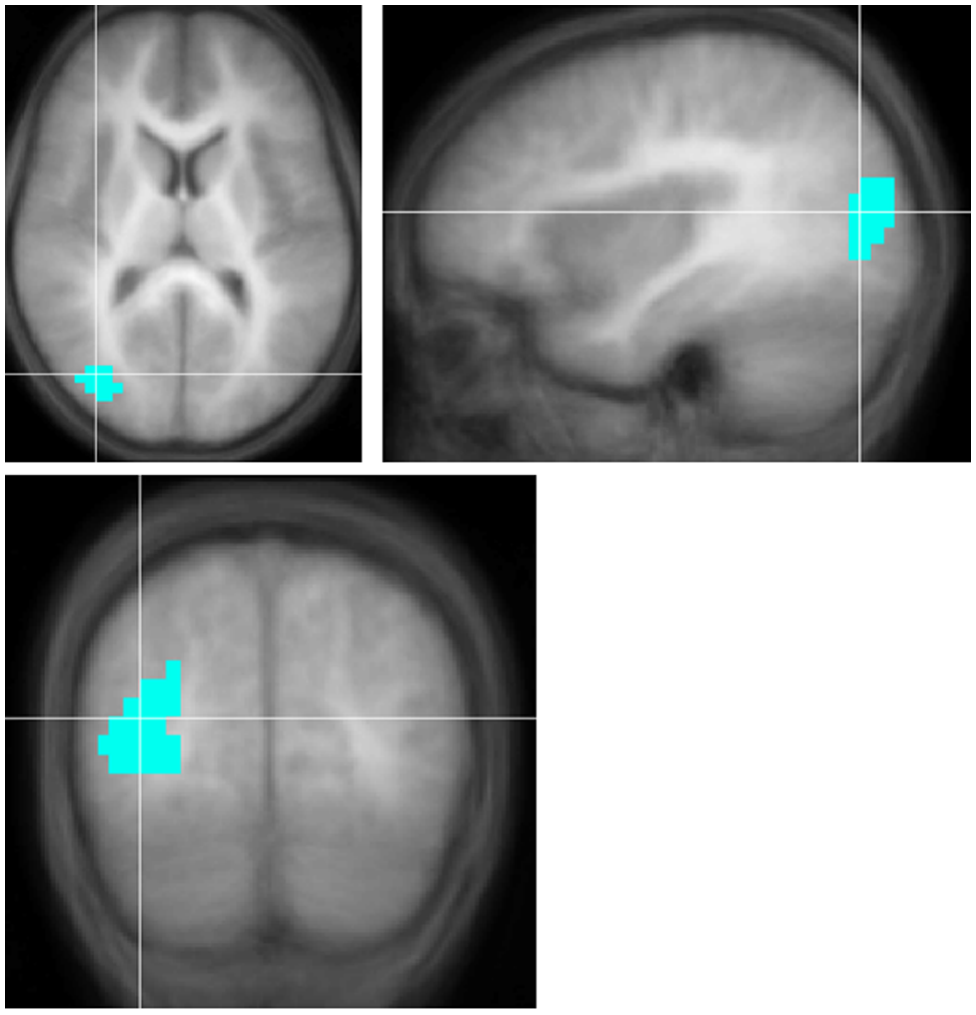
**Region with significantly *smaller* functional activation in children with severe-to-profound unilateral hearing loss for the fMRI audio–visual paradigm of a modified “Token Test” (described in more detail in the text) compared to normal-hearing controls.** Images in radiologic orientation. Slice locations (Talairach coordinate system): *Z* = +12 mm (Axial); *X* = +34 mm (Sagittal); *Y* = -71 mm (Coronal).

Children with UHL displayed smaller activation (**Figure 1**; **Table 4**) in a region encompassing the right inferior temporal, middle temporal, and middle occipital gyri (BA 19/37/39) for the contrast of all sentences vs. control, when analysis was restricted to children with severe-to-profound UHL, or children with right UHL. No significant differences were seen for the contrast of complex sentences vs. control or simple sentences vs. control.

**Table 4:** Regions with activation or deactivation differences between children with unilateral hearing loss (UHL) and normal-hearing (NH) children for the audio–visual “modified token” task.

Region	BA	Coordinates	#Voxels
**Activation in children with severe-to-profound UHL < NH children**
Right inferior temporal gyrus/middle temporal gyrus/middle occipital gyrus	19/37/39	31,-76,10	65
**Activation in children with UHL > NH children**
Left superior temporal gyrus	22	-40,-21,3	68
***Deactivation* in children with UHL < NH children**
Posterior cingulate/precuneus	31/7	-3,-40,27	395
Medial prefrontal	32/9	3,49,19	248
Left prefrontal	9	-21,31,43	106

*Coordinates in Talairach space.*

### REGIONS WITH GREATER ACTIVATION IN CHILDREN WITH UHL

**FIGURE 2:**
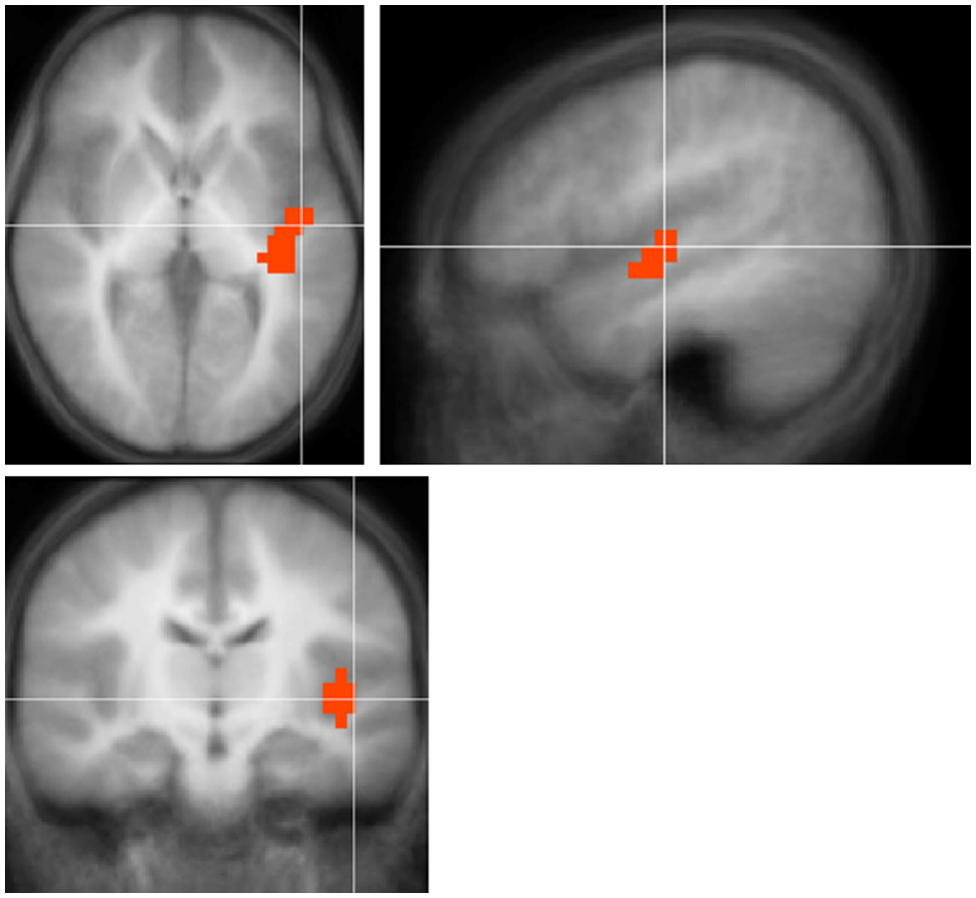
**Region with significantly *greater* functional activation in children with unilateral hearing loss for the fMRI audio–visual paradigm of a modified “Token Test” (described in more detail in the text) compared to normal-hearing controls.** Images in radiologic orientation. Slice locations (Talairach coordinate system): *Z* = +2 mm (Axial); *X* = -46 mm (Sagittal); *Y* = -12 mm (Coronal).

Children with UHL displayed greater activation (**Figure 2**; **Table 4**) in the posterior aspect of the left superior temporal gyrus for the contrast of all (simple and complex) sentences vs. control, and the contrast of complex sentences vs. control, though not for the contrast of simple sentences vs. control. The contrast of all sentences vs. control maintained significance when analysis was restricted to children with severe-to-profound UHL, though not when analysis was restricted to children with UHL of greater than 2 years duration, children with left UHL, or children with right UHL.

### REGIONS WITH SMALLER *DEACTIVATION* IN CHILDREN WITH UHL

**FIGURE 3:**
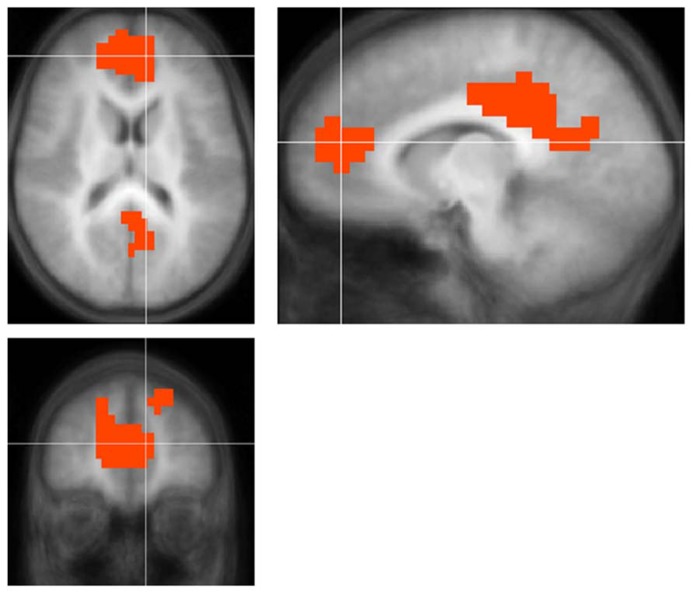
**Regions with significantly *smaller* functional *deactivation* in children with unilateral hearing loss for the fMRI audio–visual paradigm of a modified “Token Test” (described in more detail in the text) compared to normal-hearing controls.** Images in radiologic orientation. Slice locations (Talairach coordinate system): *Z* = +16 mm (Axial); *X* = -9 mm (Sagittal); *Y* = +47 mm (Coronal).

For all contrasts and comparisons (**Figure 3**; **Table 4**) children with UHL displayed smaller deactivation in posterior and anterior DMN regions, indicating that this phenomenon is ubiquitous among the population with UHL-SN.

### REGIONS WITH GREATER *DEACTIVATION* IN CHILDREN WITH UHL

No regions were found with greater deactivation in children with UHL, with the exception of the right motor region when analysis was restricted to children with right UHL (data not shown).

### COMPARISON BETWEEN CHILDREN WITH LEFT AND RIGHT UHL

No differences in activation or de-activation between children with left and right UHL-SN were found, except for deactivation differences in the right primary auditory cortex, related to listening to the 440 Hz tone during the control task (data not shown).

### *POST HOC* ANALYSES

No significant difference was found between the residual variance from scans acquired on the Siemens scanner vs. scans acquired on the Philips scanner for any of the ROIs examined [max. *F*(20,22) = 1.57, *p* > 0.25], indicating results were not biased by failing to model different variances dependent on scanner. No significant effect was found for side of hearing loss for the region with activation differences in the left posterior superior temporal gyrus from **Figure 2** [*T*(14) = -0.68, *p* > 0.5].

## DISCUSSION

This functional imaging study investigates differences in the neural correlates of cross-modal processing in children with UHL. The children in this study were of normal overall intelligence and demonstrated no other signs of neurodevelopmental disorders, minimizing possible problems related to transferability of results. This study, the first of its kind, provides unique insights into the effects of unilateral sensory deprivation on brain development.

Agreeing with our primary hypothesis, we found significantly less functional activation in secondary visual processing regions in the right hemisphere (BA 19/37/39) in children with UHL. An increasing body of evidence argues against a strictly hierarchical view of multisensory integration and demonstrates multimodal interactions in cortical regions previously thought to be unimodal [see (Kayser and Logothetis, 2007) for a review] including secondary visual processing areas. In fact, direct connections from primary and secondary auditory areas, into primary and secondary visual areas, have been shown in monkeys via tracer studies (Falchier et al., 2002; Rockland and Ojima, 2003; Wang et al., 2008). Our results suggest that development of these connections is affected by the sensory deprivation stemming from UHL. However, it should be noted this result was significant only when analysis was restricted either to children with severe-to-profound UHL-SN, or children with right UHL-SN. Children with only moderate UHL-SN still receive some input from the hearing-impaired ear, which likely ameliorates downstream effects on cognitive processes. Many studies (Bess and Tharpe, 1986; Bess et al., 1986; Oyler et al., 1987; Jensen et al., 1989) have shown differential effects on left vs. right UHL-SN for children at least until age 11 or 12, with worse language and receptive speech outcomes for children with right UHL-SN, since the input from the left ear is predominantly processed in the right auditory cortex and must therefore traverse interhemispherically to the language processing centers, which are in the left hemisphere for most individuals.

Children with UHL-SN also displayed greater activation in the left superior temporal gyrus in the region of Heschl’s gyrus. One might hypothesize this to be a function of side of hearing loss, as changes have been reported in the dendritic growth within the contralateral auditory cortex of unilaterally deafened rabbits (McMullen et al., 1988). However, this difference was no longer significant when analyses were restricted to either children with left or right UHL-SN (due to insufficient power), and *post hoc* analysis on the children with UHL-SN demonstrated no significant effect of side of hearing loss. Interestingly, the results are no longer significant when analysis was restricted to children with UHL-SN over 2 years duration. This may indicate that this finding is only a short-term effect, which could be related to lack of complete reorganization of auditory pathways, although a likely alternative hypothesis is that this may reflect more effort in low-level processing of auditory stimuli; the laterality of this effect may reflect the greater contribution of the left hemisphere to the kinds of rapid changes in the acoustic signal that are characteristic of speech (Hickok and Poeppel, 2004). A further intriguing possibility is that, as visual processing is affected by UHL, this difference also affects auditory processing in a feedback loop, as auditory processing in primary and secondary auditory areas is known to be modulated by visual stimuli (Kayser and Logothetis, 2007; Kayser et al., 2007, 2008).

Relating to our exploratory hypothesis, we found that children with UHL-SN displayed significantly less *deactivation* in posterior (posterior cingulate/precuneus) and anterior (medial pre- and orbitofrontal) DMN regions, regardless of the specific type of sentence (complex or simple), and independent of side of hearing loss. While the DMN is typically classified as a “resting-state” network, it was initially identified as a network found to deactivate during the performance of cognitive tasks; the amount of deactivation is a function of the cognitive demand and is also related to task performance (Tomasi et al., 2011; Vannini et al., 2011; Duan et al., 2012; Gilbert et al., 2012; Prakash et al., 2012). DMN activity is thought to reflect self-referential activity; when insufficiently suppressed during the performance of a demanding cognitive task, it renders the participant susceptible to mind-wandering (McKiernan et al., 2006; Mason et al., 2007).

These results lead us to hypothesize that the degree to which the DMN deactivation is affected by UHL may be a key factor in subsequent outcomes. The academic and behavioral deficits seen in children with UHL may be mediated by deficiencies in the DMN, indicating children with UHL are insufficiently suppressing self-referential activity during performance of demanding cognitive tasks. The results in the DMN maintained significance whether analysis was performed over all participants, or restricted to participants with verified long duration (> = 2 years) of hearing loss, severe-to-profound hearing loss, or left or right hearing loss, indicating this phenomenon to be ubiquitous over the population. Thus, ameliorating noisy backgrounds via FM amplification (Kenworthy et al., 1990; Updike, 1994) may be a necessary, but not sufficient, condition for an optimized intervention strategy in children with UHL. Remediation strategies for executive function deficits (associated with DMN deficiencies) may also be a necessary component. Such strategies may include behavioral, but also possibly pharmacological components, as such have been proven successful in remediation of math and language deficits in pathologies such as ADHD which also are associated with DMN deficits (Douglas et al., 1986; Benedetto-Nasho and Tannock, 1999; Hechtman et al., 2004; Silva et al., 2005; Grizenko et al., 2006; Rubinsten et al., 2008).

It is interesting to note that differences in functional activation and de-activation occur in a task involving speech in quiet. A significant effect of UHL was not found either on in-scanner task performance or out-of-scanner performance for the SCAN-C subtests involving speech in quiet (e.g., compressed sentences and filtered words; Our task design emulates speech in quiet, rather than speech in noise, as the background level of the MRI-compatible sound system was measured at < 10 dB SPL and stimuli were presented during completely silent scanner intervals). Our results therefore confirm that UHL affects development of neural substrates used for cross-modal modulation even for tasks such as speech in quiet where performance is not directly affected. However, these differences may result in downstream effects on other behavioral outcomes.

Accordingly, in the broader picture, this study demonstrates that a change in the transmission of sound to the brain from binaural to monaural input can have a global influence on the development of brain networks related to higher-order cognitive function. This result may inform the ongoing debate concerning other disorders that involve subtle changes involving audition. For example, whether APD in children should be viewed as more of a sensory, or a higher-order cognitive, deficit is a matter of active debate (Moore et al., 2010). However, our results suggest that this may be a matter of “both-and” rather than “either-or”: subtle auditory deficits such as those hypothesized to exist in APD may themselves contribute to a spectrum of neurocognitive deficits. Thus APD (which may be the result of a subtle deficit in connectivity between the medial geniculate and the auditory cortex; Schmithorst et al., 2013) may result in downstream influences on the later development of cognitive function, including executive function and attention, similar to what was shown in this study with UHL. Future research will investigate this hypothesis in more detail.

Deficiencies in DMN deactivation during the performance of demanding cognitive tasks has been demonstrated in various neuropathologies including math disability (Davis et al., 2009), autism (Christakou et al., 2013), schizophrenia (Das et al., 2012), and ADHD (Christakou et al., 2013). Thus, this study provides preliminary evidence of a neurobiological substrate common to all these deficits, and includes in this grouping the neurological sequelae of UHL. An important question for future research is to investigate whether DMN deficiencies are themselves a fundamental neurobiological etiology of various disorders, and whether the severity of DMN effects relates to the spectrum of behavioral manifestations and regional brain function of these heterogeneous disorders. DMN deficiencies could be a common etiology of a spectrum of behavioral deficits that vary in individuals due to differences in and interactions with environment [see (Menon, 2011) for a review]. The alternative explanation is that DMN deficiencies are a result of other, more fundamental neuropathologies, in a similar manner as we propose is the case for a sensory deficit such as UHL. Even in this case, however, strengthening of the DMN network may result in improved outcomes. This has been shown, for instance, in improved performance in math processing after stimulant medication in children with ADHD (Douglas et al., 1986; Benedetto-Nasho and Tannock, 1999; Hechtman et al., 2004; Silva et al., 2005; Grizenko et al., 2006; Rubinsten et al., 2008).

A possible limitation of the study is that the NH and UHL cohorts are not precisely matched on levels of overall cognitive function. While the UHL cohort is in the normal range, the NH children display approximately 2/3 of a standard deviation higher mean IQ scores. This was taken into account by covarying for full-scale IQ in all neuroimaging analyses. Additionally, we are unable to determine, in this cohort, whether hearing loss was congenital or acquired. This limitation is present in all studies of UHL before the advent of universal newborn hearing screening (UNHS); unfortunately, UNHS in Ohio began only in 2004. Without such procedures in place, UHL in children with a congenital loss is typically only detected after the start of school, at approximately 5 years of age. While it is likely that most participants in our study with a verified long duration of hearing loss had an acquired loss, as is the case for the majority of individuals with UHL, we cannot rule out that some of the participants with verified long duration of hearing loss may in fact have a congenital loss. Nevertheless, our results suggest that the DMN is affected even by acquired UHL; this result is consistent with previous studies suggesting acquired UHL is associated with language, speech, and behavioral deficits. Moreover, as the DMN itself is in place at birth (Doria et al., 2010), even for individuals with a congenital loss UHL will be affecting the relation of the DMN to cognitive processes, and not its original development. Finally, we are unable to rule out the possibility, however unlikely, that the differences in brain function seen are not a direct result of UHL but are, instead, a result of the cause of UHL (such as genetics or infection). A future longitudinal study would be necessary to definitively resolve this question.

In conclusion, our results show altered neurophysiology in children with UHL for cross-modal modulation as well as a deficiency in deactivation of the DMN during audio–visual tasks. These results strongly suggest that one good ear is insufficient to promote the development of normal cognitive function. This physiological signature may underlie the poor academic and behavioral outcomes associated with UHL.

## Conflict of Interest Statement

The authors declare that the research was conducted in the absence of any commercial or financial relationships that could be construed as a potential conflict of interest.
